# A Comprehensive Analysis on Abundance, Distribution, and Bionomics of Potential Malaria Vectors in Mannar District of Sri Lanka

**DOI:** 10.1155/2019/1650180

**Published:** 2019-03-12

**Authors:** Nayana Gunathilaka, Menaka Hapugoda, Rajitha Wickremasinghe, Wimaladharma Abeyewickreme

**Affiliations:** ^1^Department of Parasitology, Faculty of Medicine, University of Kelaniya, Sri Lanka; ^2^Molecular Medicine Unit, Faculty of Medicine, University of Kelaniya, Sri Lanka; ^3^Department of Public Health, Faculty of Medicine, University of Kelaniya, Sri Lanka

## Abstract

**Background:**

A detailed knowledge of the distribution of the malaria vectors in Mannar district of Sri Lanka has not been studied after 1927. Past records indicated the presence of only seven species of anophelines, namely,* An. culicifacies, An. subpictus, An. barbirostris, An. peditaeniatus, An. nigerrimus, An. Jamesii,* and* An. maculatus*. There have been many changes in terms of distribution of* Anopheles* in the district over time.

**Methods:**

Entomological surveillance was conducted on a monthly basis, comprising indoor hand collection, window trap collection, cattle-baited net collection, cattle-baited hut collection, and larval survey from June 2010 to June 2012 in 12 study areas under three entomological sentinel sites. The relationship between seven abiotic variables of the breeding habitats was measured. Pearson's correlation coefficients were used to determine the associations between climatic variables and anopheline densities.

**Results:**

A total of 74,181 mosquitoes belonging to 14* Anopheles* species were recorded.* An. subpictus* was the most predominant species from all techniques representing 92% (n=68,268) of the total anopheline collection. However,* Anopheles culicifacies* was not recorded from any site during the study period. Larval surveys identified 12 breeding habitat categories including waste water collections, lagoon water collections, and drains which were not recorded as breeding habitats by previous studies. The mean dissolved oxygen level of waste water collections was 3.45±0.15 mg/l. The mean salinity and conductivity of lagoon water collections were 21105±1344 mg/l and 34734±1974 *μ*s/cm, respectively.

**Conclusion:**

The present study provides the updated knowledge on anopheline distribution and vector bionomics. Therefore, documentation of the current knowledge would be useful for learners and health authorities to design appropriate vector control measures in the prevention of reintroduction of malaria.

## 1. Background

Malaria is a vector borne disease transmitted by adult female mosquitoes of the genus* Anopheles*. This disease is endemic in tropical and subtropical regions of the world [1]. It was one of the main public health issues in Sri Lanka in the past. The most prevalent cause of malaria was* Plasmodium vivax* (70%) while the rest of the cases were caused by* Plasmodium falciparum* [2]. There have been no local cases of malaria recorded from the country since October 2012. This remarkable success was accomplished rapidly and largely during a protracted civil war [3].

Sri Lanka reached the “malaria free” certificate from the World Health Organization (WHO) in September 2016. However, it remains vulnerable to reintroduction and transmission of malaria due to the continuous influx of imported malaria cases through travelers to the country and malaria receptivity being high in most parts of the country [4]. The risk of onward transmission by individuals acquiring infection will depend on the presence of vectors, climatic and other socioenvironmental conditions suitable for transmission within the country.

According to the disease history, Sri Lanka almost reached the elimination status once before exactly five decades ago with only 17 cases reported in 1963 and subsequently losing the grip which led to resurgence of malaria mounting to over 400,000 cases in 1967/68. Withdrawal of malaria control measures and weakened surveillance were identified as some of the factors influencing the above situation [4]. Therefore, it is imperative to have a proper understanding of imported malaria and the prevalence and distribution of the vectors to plan out effective preventive strategies accordingly in order to prevent the reintroduction of malaria.

To date, 24 anopheline species have been reported from Sri Lanka with the addition of* Anopheles jeyporiensis* in 2015 and* Anopheles stephensi* in 2017 [5, 6]. Of them* Anopheles culicifacies* was regarded as the only malaria vector in the country until the early 1980s. However, enzyme-linked immunosorbent assay (ELISA)-based evidence has shown a large number of anopheline species infected with malaria parasites. These include* Anopheles aconitus*,* Anopheles annularis*,* Anopheles barbirostris*,* Anopheles nigerrimus*,* Anopheles pallidus*,* Anopheles subpictus*,* Anopheles tessellatus*,* Anopheles vagus*, and* Anopheles varuna* [7].

Over three decades of civil unrest, the conflict situation has had detrimental effects on vector control activities and management of malaria in Mannar district, which was previously regarded as a malaria endemic region in Sri Lanka. Detailed entomological investigations have been carried out in Mannar island (Northern Province) in 1913 [8] and 1927 [9]. However, there is no detailed study available after 1927 in this district as a published material. The distribution of* Anopheles*, and more especially their numerical availability, is a subject of considerable importance in connection with the prevalence and probable dissemination the diseases. The mere presence of anopheline mosquitoes or even their presence in large numbers may be of comparatively little economic importance if the species concerned are not efficient carriers of the disease.

There is comparatively little information available on the extended distribution of the indigenous* Anopheles* species, although observations on the various species occurring in certain towns had been made from time to time [10–14]. Some people in the district of Mannar visit nearby countries like India and Pakistan which are still suffering from the disease severely. Hence, these people could be vulnerable to malaria at their visit any time and even from them a reintroduction of malaria could occurr in the country due to imported cases with the presence of malaria vectors in these areas [3].

This study is intended as the second published document after 1927 which is dealing with the distribution and numerical prevalence of the various anopheline species, their seasonal periodicity, bionomics, breeding places, and their relative importance as malaria transmitting vectors in the district of Mannar, Sri Lanka.

## 2. Methods

### 2.1. Study Area

District of Mannar is located in the Northern Province of Sri Lanka. It has an area of 2,002 km^2^ with 106,235 human population. The average temperature and mean annual rainfall of the study area are 24.6-31.5°C and 1,051 mm, respectively. From the climatic point of view, the study regions are conducive to malaria epidemics and can be supported by the presence of malaria vectors,* Anopheles culicifacies, Anopheles subpictus, Anopheles annularis, Anopheles varuna, *and* Anopheles tessellatus.* Moderate level house types which are made of unplastered brick walls with tiled or asbestos roofs are predominant. Farming and fishing are the major livelihoods of the people living in this district.

### 2.2. Sentinel Sites and Localities in Study Areas

**Figure 1:**
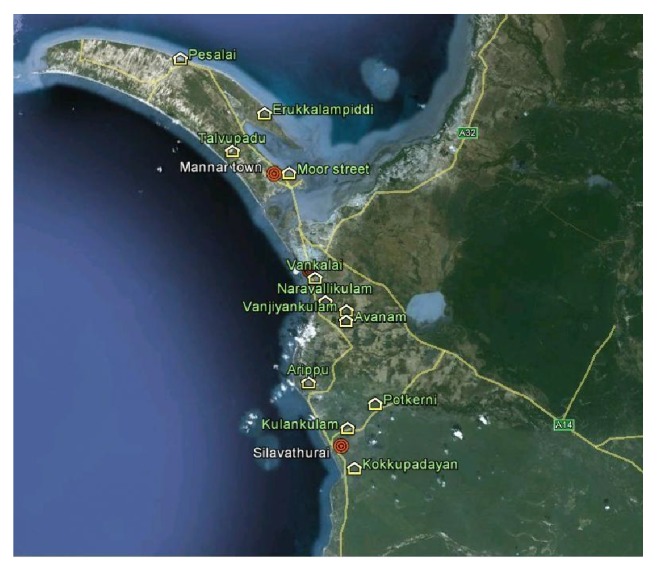
Map indicating sentinel sites and localities in Mannar district.

A sentinel site was defined as an area, where malaria transmission risk is present over a period of time or/and where increased potential for vector breeding is well established. High risk area may be a previously malaria risk area or an epidemic prone area. Three sentinel sites (Mannar Town, Vankalai, and Silawathura) were identified within a radius of 30 km and each site was further subdivided into four localities (within 5-30 km) to ensure full coverage of the sentinel site during the surveillance (Figure 1).

### 2.3. Entomological Surveillance

Entomological surveys were conducted on a monthly basis from June 2010 to July 2012 using five standard sampling methods according to WHO standard techniques for anopheline mosquitoes [15].

### 2.4. Indoor Hand Collection (HC)

Indoor hand collections were conducted in randomly selected houses in each locality using standard mouth aspirators. Mosquitoes were collected from a minimum of 180 houses per month in a sentinel site (45 from each locality). Collections were made during the morning (06.00-08.30 hrs) by two vector collectors spending a maximum of ten minutes per house. Bedrooms, preferably with complete walls and the highest number of persons slept the previous night, were given priority.

### 2.5. Window Trap Collection (WTC)

Two mosquito window (exit) traps were fixed in a sentinel site for 16 nights per month. The following day mosquitoes were collected by two trained persons.

### 2.6. Cattle Baited Net Collection (CBNC)

The trap was made out of white cotton drill (3 m × 3 m x 1.5 m) with net windows (2 m × 1 m) on sides and erected using a strong centre pole of two meter height and four side sticks of the same height. The trap was set about 50 m away from the houses and away from the place, where cattle are usually tethered or herded during the night. A distance of 15-25 cm gap was allowed between the lower edge of the net and the ground, enabling mosquitoes to enter in. At sunset a cattle introduced into the trap in the evening and tethered to the pole fixed to the mid of the hut. The cattle removed at dawn for collecting the mosquitoes. All anophelines resting inside the trap were collected.

### 2.7. Cattle Baited Hut Collection (CBHC)

A standard hut was constructed in each locality. The size of the hut suited the size of the cattle bait and was approximately 2 m x 1.25 m x 1.25 m. It was made of sticks and poles and thatched with woven cadjan. At sunset, a calf was tethered to a strong pole inside the hut with no windows. A removable door made out of sticks and cadjan was fitted to the hut to facilitate the movement of the calf and the collector in and out of the hut. A space of about 10-15 cm between the ground and the cadjan thatched wall and about a five cm space between the roof and the wall were left for the movement of mosquitoes. All anophelines resting inside the hut were collected on the following day.

### 2.8. Larval Survey (LS)

All potential breeding habitats were identified in all 12 localities through a preliminary survey conducted for a period of one month prior to the research study and certain fixed and temporary breeding places were identified for the larval survey. Larval surveys were conducted by standard dipping method using ladles (250 ml capacity).

### 2.9. Sample Identification

#### 2.9.1. Adults

Live mosquitoes collected from HC, WTC, CBNC, and CBHC were anaesthetized with 70% chloroform and transferred into Petri dishes lined with wet filter papers. Anaesthetized mosquitoes were identified by an achromatic magnification lenses (x10) using standard morphological keys prepared for the Sri Lankan* Anopheles* adult mosquitoes [16, 17].

#### 2.9.2. Immature Stages

Mosquito larvae were placed individually in a depression microscopic slide with a minimum amount of water and identified under a light microscope (Olympus Optical Co. Ltd., Tokyo) with an objective (x 10). Stages III and IV instar larvae collected from the field and I & II stages reared to III^rd^ stage in the field station were identified using standard morphological keys prepared for the Sri Lankan* Anopheles *larvae [5].

### 2.10. Physicochemical Properties of Vector Breeding Habitats

#### 2.10.1. Collection of Water Samples

Three water samples were collected into glass collecting bottles separately from each breeding habitat concurrently with the collection of mosquito immature, between 09:00 and 12:00 hr on each sampling day.

#### 2.10.2. Detection of Water Quality Parameters

Seven abiotic variables: temperature, hydrogen ion concentration (pH), conductivity, Total Dissolved Solids (TDS), turbidity, salinity, and dissolved oxygen (DO) were measured on site at the time of collection, temperature (portable meter, Hach SenSION TM), pH (portable meter, Hach SenSION TM), and DO (digital meter EUTECH Dowp 300/02K). Conductivity, TDS, and salinity were also measured (Hach SenSION TM multiprobe meter).

#### 2.10.3. Collection of Climatic Data

Monthly climatic data including rainfall (RF), temperature (MT), and relative humidity (RH) of the Mannar district monitored at various locations were obtained from the Department of Meteorology, Colombo, Sri Lanka.

### 2.11. Data Analysis

#### 2.11.1. Entomological Survey

The density of each mosquito species collected by CBHC, CBNC, and WTC was calculated as per trap densities (Number of mosquitoes from each species/ Total number of traps), HC as density per house (Number of mosquito from each species/Total number of houses surveyed) densities of the anopheline larvae were calculated as density per 100 dips {(Number of mosquitoes from each species/Total number of dips) x 100}. These were interpreted in percentage and presented in tables. Pearson's correlation coefficients were used to determine the associations between climatic variables and anopheline densities.

#### 2.11.2. Seasonal Dynamics and Distribution of Larvae

Seasonal dynamics of mosquito larvae populations in the sampling sites was analyzed using the following factors [18].

Distribution was determined as the percent of sampling sites in which a species was noted, according to the formula:

(1) $$\begin{array}{c} C=\frac{n}{N}\cdot 100\% \end{array}$$

where

C is distribution, n is number of sites of the species, and N is Number of all sites.

Five distribution classes were defined as C1 - sporadic appearance (constancy 0 - 20%), C2 - infrequent (20.1 - 40%), C3 - moderate (40.1 - 60%), C4 - frequent (60.1 - 80%), and C5 - constant (80.1 - 100%) based on the recommendations of Banaszak and Winiewski [18].

The density of mosquito larvae in each breeding habitat was calculated using the following formula [18]:

(2) $$\begin{array}{c} D=\frac{1}{L}\cdot 100\% \end{array}$$

where

D is Density, l is Number of specimens of each mosquito species, and L is Number of all specimens.

Based on the calculations, the three density classes were accepted as Satellite species (D < 1%), Subdominant species (1< D <5%), and Dominant species (D > 5%).

#### 2.11.3. Physicochemical Properties in Different Breeding Habitats

Significance in the variations in physicochemical properties in different breeding habitats were examined by One-Way Analysis of Variance (One-Way ANOVA) followed by Turkey's pair wise test using MINITAB 17.0 software package. Relationships between abundance and physicochemical variables in breeding habitats were examined by Pearson's correlation analysis. Values with P <0.05 were considered as statistical significant correlations.

## 3. Results

### 3.1. Entomological Surveillance

A total of 74,181 mosquitoes belong to 14* Anopheles* species were recorded. The majority (33.1%) of adults were collected by cattle baited collections (CBHC and CBNC). Larval surveys represented 50.9% (37,788/74,181) of the total* Anopheles* detected from the survey. The overall results of the mosquitoes collection made by five sampling techniques are illustrated in Table 1.

*Anopheles subpictus* was the predominant species detected from HC (98.2%, n=9,854), WTC (98.6%, n=1,792), CBHC (86.6%, n=11,170), CBNC (78.1%, n=9,101), and LS (96.2%, n=36,351). However,* Anopheles culicifacies* was not recorded from any site during the study period. The relative abundance of anopheline mosquitoes encountered during the study is given in Table 2.

Immature stages were collected from 12 types of breeding habitats at monthly intervals from each locality. The major habitat categories were tank margin, wastewater collection, water storage tank, field canal, main canal, paddy field, pond, built well, cemented tank, lagoon water collection, burrow pit, and rain water pool (Table 3). Built wells and waste water collections were conducive for anopheline breeding.

Of the species encountered from larval surveys,* An. subpictus* (96.2%) was the predominant (n= 36,351) followed by* An. peditaeniatus *(1.47%, n=557),* An. barbirostris* (1.23%, n=463),* An. nigerrimus* (0.75%, n=285),* An. varuna* (0.19%, n=74),* An. barbumbrosus* (0.1%, n=38),* An. vagus* (0.03%, n=12),* An. pallidus* (0.01%, n=4),* An. jamesii* (0.05%, n= 2), and* An. pseudojamesi* (0.05%, n=2).

According to the density criterion,* An. subpictus* was observed as satellite species (D > 5%).* An. peditaeniatus* and* An. barbirostris* were recorded as subdominant species (1< D <5%). All other species were noted as satellite species (D < 1%). Only* An. subpictus* can be regarded as constant according to distribution (C= 80.1 - 100%).* An. nigerrimus* was observed as a frequent species (C= 60.1 – 80%).* An. peditaeniatus *and* An. barbirostris *were identified as moderate species (C= 40.1 - 60%). Only* An. pallidus* was recorded as an infrequent species (20.1 - 40%). All other species belonged to the distribution group of sporadic appearance (Table 4).

### 3.2. Seasonal Variation and Correlation of Anopheline Densities with Climatic Factors

**Figure 2:**
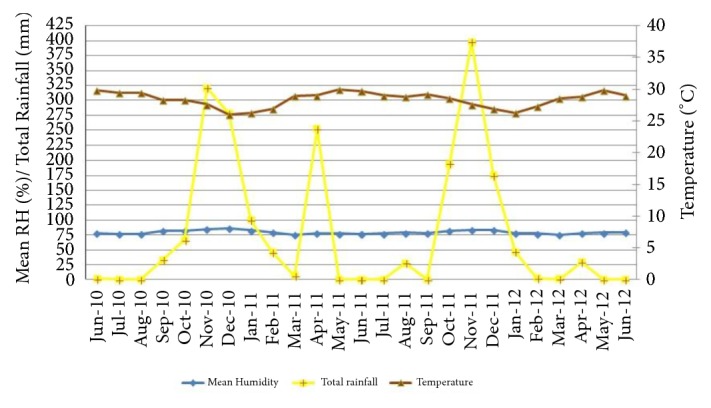
Climatic details in the district of Mannar during the study period.

The highest rainfall was observed during the months of October to December. The mean monthly relative humidity was over 75%. The mean monthly temperature varied from low 26°C to high 29.9°C. There was a positive significant correlation between RF of current month with RH of current month (*r* = 0.83; P=0.01), one of the previous month (*r* =0.52; P=0.01) and two months (*r* =0.49; P= 0.05) lag period. The variation of climatic factors in the district of Mannar from June 2010 to June 2012 is given in Figure 2.

### 3.3. Indoor Resting Anophelines Collected by HC

**Figure 3:**
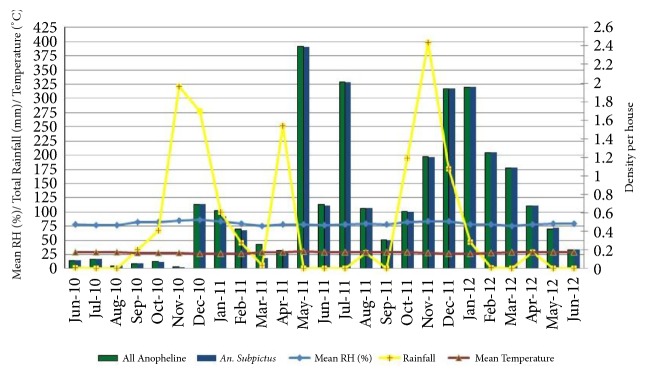
Density of* An. subpictus* and all anophelines collected by HC with climatic variables.

The density of all anophelines and* An. subpictus* was high during the monsoonal rains from May to July and November to January of the following year (Figure 3).* An. subpictus* density was positively correlated with TM of the current month (*r* =0.25; P=0.21) and one month lag period (*r *=0.12; P=0.54). Relative humidity having a two-month lag period was positively correlated with* An. subpictus* (*r *=0.13; P=0.54) and all anophelines (r =0.14; P=0.50), though not significantly (Table 5).

### 3.4. Indoor Frequenting Anophelines Collected by WTC

**Figure 4:**
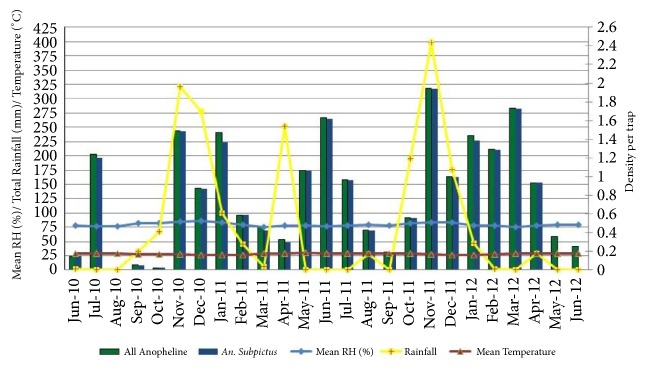
Density of* An. subpictus* and all anophelines collected by WTC with climatic variables.

The anopheline densities by WTC were high during monsoonal rains from May to July and November to January periods in the district.* An. subpictus* was the predominant species among anophelines fauna and the highest density was observed in the month of November in both 2011 and 2012 when there was high rainfall (Figure 4). Positive correlations were observed between* An. subpictus* density with RF of the current month (*r* =0.18; P=0.93), having one* (r* =0.15; P=0.46) and two months (*r* =0.10; P=0.63) lag periods (Table 5).

### 3.5. Anophelines Collected by CBHC

**Figure 5:**
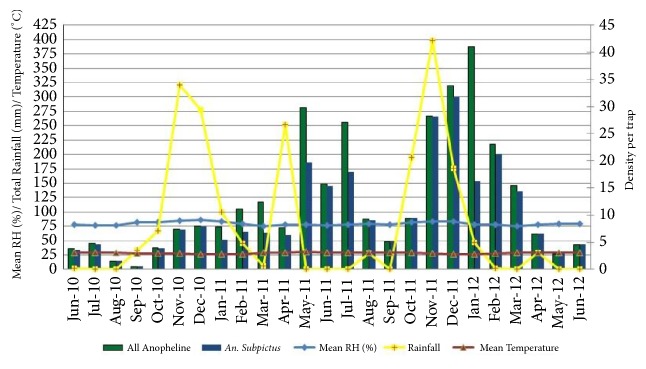
Density of* An. subpictus* and all anophelines collected by CBHC with climatic variables.

The density of all anophelines and* An. subpictus* was high during the monsoonal rains from May 2011 to July 2011 and November 2011 to January 2012, which was similar to the pattern of mosquitoes detected from HC (Figure 5).* An. subpictus* was positively correlated with the RF of the current month (*r* =0.31; P=0.13) and having two-month lag period (*r *=0.09; P=0.65), though not significantly correlated. There was a positive correlation between* An. subpictus* with RH of the current month (*r* =0.22; P=0.29), having one-month (*r *= 0.23; P=0.27) and two-month lag period (*r *=0.21; P=0.33), though not significantly (Table 5).

### 3.6. Outdoor Resting Anophelines by CBNC

**Figure 6:**
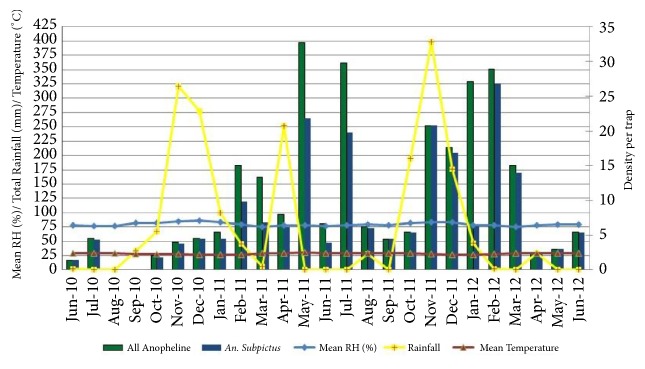
Density of* An. subpictus* and all anophelines collected by CBNC with climatic variables.

The variations in outdoor resting of all anophelines and* An. subpictus* mosquito densities collected by CBNC collections were similar to the pattern observed from CBHC. The highest densities were observed from May to June and from November to February of the following year (Figure 6). All anophelines were positively correlated, though not significantly, with RF having a one-month (*r* =0.28; P=0.17) and a two-month lag period (*r* =0.24; P=0.26). Relative humidity was positively correlated with the current month, having one and two lag periods of* An. subpictus* and all anopheline encountered (Table 5).

### 3.7. Larval Population

**Figure 7:**
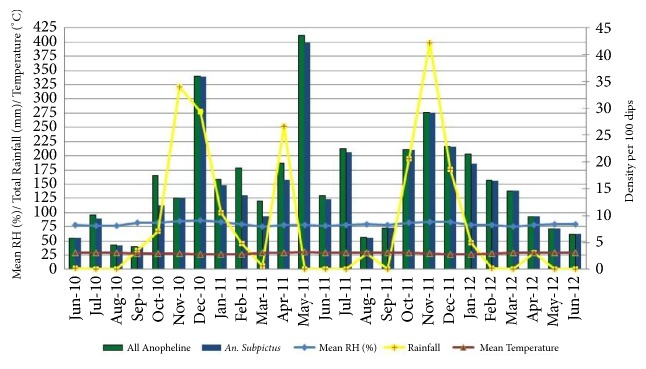
Density of* An. subpictus* and all anophelines collected by LS with climatic variables.

Larval densities of all anophelines were higher during the monsoonal rains (May to July and October to December) including* An. subpictus* (Figure 7). There was a significant positive correlation between RF of the current month with the densities of all anophelines (*r* =0.411; P=0.04) and* An. subpictus* (*r* =0.416; P=0.03). Positive significant correlations were also observed between RH of the current month* (r* =0.44; P=0.02) and one month lag period (*r *= 0.45; P=0.02) with larval density of* An. subpictus* (Table 5).

### 3.8. Physicochemical Properties of Anopheline Breeding Habitats

Mosquito larvae were found in 12 types of water collections. In total, 1,374 breeding habitats were analyzed for seven physicochemical parameters.* An. subpictus* was predominant in all habitat categories. The mean physicochemical characteristics of water in breeding habitat types are given in Table 6. The breeding of* An. subpictus* was more conducive with high conductivity, salinity, and TDS dissolved oxygen concentrations in breeding habitats of waste water collections, water storage tanks, and paddy fields which were significantly different (P < 0.05). Hydrogen ion concentration in tank margins and waste water collections, ponds and lagoon water collections was not significantly different. There was no difference in the TDS levels in field canals, paddy fields, and burrow pits. The TDS levels of waste water collections, water storage tanks, ponds, lagoon water collections, and cement tanks were significantly different (P< 0.05). The salinity in main canals, built wells, lagoon water collections, and rain water pools was significantly different.

## 4. Discussion

Comparison of the anopheline mosquito population of two surveys in both the past (1924–1927) and the present (2010–2012) clearly shows that there are some significant changes in the abundance of mosquito species. The present study demonstrates that the mosquito replacement after 86 years of time is due to advancement in the taxonomy, environmental changes caused by urbanization, resettlements, and development projects in the form of increased mosquito breeding habitats. According to the past records, larvae and adults of seven species of* Anopheles, *namely,* An. culicifacies, An. subpictus, An. barbirostris, An. peditaeniatus, An. nigerrimus, An. Jamesii,* and* An. maculatus *were identified from various parts of the Mannar district, but adults were scarce and difficult to find. Of them,* An. culicifacies* was the most abundant species in dwellings, and dissections of captured females proved it to be actively engaged in the transmission of malaria [8, 9].

The present study encountered 14 anophelines including* An. annularis*,* An. barbumbrosus*,* An. kawari*,* An. pallidus*,* An. pseudojamesi*,* An. tessellatus*,* An. Vagus,* and* An. varuna*, in addition to the above list of species recorded in the past. The most abundant species was* An. subpictus* both in larval and adult collection methods. However,* An. culicifacies*, the established principal vector of malaria in Sri Lanka, was not found in the surveillance sites of Mannar throughout the study period.

Anopheline larval densities were correlated significantly and positively with total monthly RF. Hence, the increase in the RF of the current month may be used to predict larval densities of all anophelines including* An. subpictus* in the district of Mannar. However, a minimal larval breeding trend was detected, when the RF was above 400 mm. This phenomenon could be due to larval flush off, since high water currents and flooding have been reported to be contributing to larval deaths of* Anopheles *species resulting from the reduction in oxygen tension causing physical harm to the larvae [19].

Breeding of anophelines are positively associated with dissolved oxygen (DO) and more prominent in water bodies with high DO [20]. However, recent studies evidenced that* Anopheles* mosquitoes including* An. culicifacies* sibling species E can tolerate low DO levels [21, 22]. There was a habitat partitioning, which implies that the mosquito species share the food resources within the same habitat. This study also noted more diversity in* Anopheles* larval compositions as compared to previous studies conducted [23]. Further, the present study found a higher diversity in the* Anopheles* larval composition as compared to the previous studies conducted, especially in these areas [9]. According to the previous studies, abundant rice field, deeper depression, burrow pit, well (abundant), pool, cement tank (cisterns), and barrel (metal) were the major breeding habitat categories. However, the present study found 12 main breeding habitats categories, namely, tank margin, wastewater collection, water storage tank, field canal, main canal, paddy field, pond, built well (domestic), cemented tank, lagoon water collection, and burrow pit.

*An. subpictus* was found in some specific breeding habitats such as wastewater collections with low dissolved oxygen levels, lagoon water bodies with high salinity, and overhead/water storage tanks at residences. Therefore, this indicates that the same species can tolerate a wide range of physicochemical conditions even at the same geographical territory.

Interestingly some specimens collected especially from lagoon water collections and wastewater collections presented with some morphological features similar to* Anopheles sundaicus*,* Anopheles epiroticus,* and* Anopheles stephensi* [16, 24]. Some studies emphasize that there may be some slight differences between the specimens observed in different geographical locations and regions [25]. Therefore, detail investigations are essential in order to explore this context with the aid of molecular entomological tools.

This study opens an avenue to explore new breeding habitats of malaria vectors in the country. Therefore, this phenomenon should be further investigated, giving special attention towards quality of water in these breeding sites. There was a perennial abundance of indoor resting anophelines including* An. subpictus*. Annually, two peaks of indoor resting vector densities were observed coinciding with the monsoonal rains. The outdoor resting anopheline population also had two distinctive peaks, but these corresponded to the period following the monsoonal rains.

The entire* Anopheles* population was more abundant in monsoonal and immediate postmonsoonal rains. The highest indoor resting densities were observed in May, 2011, and January, 2012, when the RF was minimal. Further, vector densities increased significantly soon after the high peak of rains in April, 2011, and November, 2011. Particularly, December to February period was noted as the best seasons for indoor resting of* An. subpictus*.

The present study revealed that* An. subpictus* was abundant in both indoor and outdoor resting habitats. Since* An. subpictus* is the secondary vector for malaria in Sri Lanka [5, 16], this species has the capacity as the most prevalent species in indoor and outdoor resting habitats in the study areas for playing an important role in the transmission of malaria at a probable disease outbreak.

Some recent studies conducted in the country have reported that the* An. subpictus* is more preferred to rest in cadjan huts than in net traps [26–28]. The current study revealed that even though CBNC recorded the highest anophelines compared to CBHC, the percentage of* An. subpictus* recorded from CBHC was comparatively high. Therefore, the present study also found a similar finding that the* An. subpictus* are more preferred to rest in cadjan huts.

Out of all adult collection techniques, cattle baited collections seem to be more favorable for outdoor feeding population in these areas. This is due to the zoophilic nature of the malaria vectors. These study areas are mainly agricultural in nature, and there are cattle reared in almost all the houses. In addition, there are stray cattle, which attract most of the feeding anopheline population. Therefore, this might be the case that a low density of anophelines was observed by HC and WTC in the study.

In this study, more* Anopheles* adults were collected outdoors as compared to indoors. It appears that anopheline mosquitoes, including vectors of malaria, prefer resting outdoors than indoors. However, insecticide residual spraying (IRS) is commonly used as the major malaria control intervention targeting adult mosquitoes. Therefore, the tendency of resting adult mosquitoes in outdoor resting surfaces will depreciate the effectiveness of IRS as a controlling measure.

Overall, the presence of malaria vectors along with diversified breeding habitats could be a challenge for sustaining interrupted transmission and preventing reintroduction of malaria in Sri Lanka. Therefore, intensified efforts in surveillance should be encouraged and enforced to prevent reintroduction of malaria due to continuous influx of imported malaria cases through travelers to the country and malaria receptivity being high in these areas.

## 5. Conclusion

*Anopheles subpictus* is the predominant species recorded from all techniques. Composition of anopheline population in both the past (1924–1927) and the present (2010–2012) clearly shows that there are some significant changes and reduction of vector densities. Therefore, documentation of the current knowledge would be useful for health authorities to design appropriate control measures in order to prevent the reintroduction of malaria with the increase of imported cases. New breeding habitats such as wastewater collections, lagoon water collections, and wells can be served as larval reservoirs during the dry season. Therefore, presence of these habitats in close proximity to human habitats creates a high risk of malaria transmission among humans. Hence, health authorities need to be vigilant on these new habitats in vector control programmes and intensified efforts in surveillance should be encouraged to prevent reintroduction of malaria due to imported cases.

## Figures and Tables

**Table 1:** * Anopheles* collected from June 2010 to June 2012.

Sampling technique	Unit	Total no. of units	Total no. collected	Percentage (%)
HC	House	14,283	10,027	13.52
WTC	Trap	2,156	1,817	2.44
CBNC	Trap	1,097	11,650	15.70
CBHC	Trap	1,041	12,899	17.38
LS	Dip	237,660	37,788	50.94
Total			74,181	100

**Table 2:** Relative abundance of anophelinesby sampling technique in Mannar district.

*Anopheles* Species	Number and percentage (%) of mosquitoes collected by				
	LS	HC	WTC	CBHC	CBNC
	(n=37,788)	(n=10,027)	(n=1,816)	(n=12,899)	(n=11,650)
*An. annularis*	-	-	-	0.008 (1)	-
*An. barbirostris*	1.23 (463)	1.11 (114)	0.28 (5)	3.64 (470)	4.19 (489)
*An. barbumbrosus*	0.1 (38)	-	-	0.78 (101)	0.91 (106)
*An. jamesii*	0.005 (2)	-	-	-	0.017 (2)
*An. karwari*	-	-	-	-	0.009 (1)
*An. maculatus*	-	0.029 (3)	-	-	-
*An. nigerrimus*	0.75 (285)	0.55 (55)	0.66 (12)	3.39 (438)	7.06 (822)
*An. pallidus*	0.01 (4)	-	-	0.29 (38)	0.63 (73)
*An. peditaeniatus*	1.47 (557)	-	0.28 (5)	5.12 (661)	8.99 (1,048)
*An. pseudojamesi*	0.005 (2)	-	-	-	-
*An. subpictus*	96.2 (36,351)	98.2 (9,854)	98.6 (1,792)	86.6 (11,170)	78.1 (9,101)
*An. tessellatus*	-	-	-	0.015 (2)	-
*An. vagus*	0.03 (12)	0.01 (1)	0.17 (3)	0.12 (16)	0.017 (2)
*An. varuna*	0.19 (74)	-	-	0.015 (2)	0.05 (6)

**Table 3:** Breeding habitats of *Anopheles* species in the Mannar District.

Type of breeding habitats	Species									
	*An. subpictus*	*An. peditaeniatus*	*An. pallidus*	*An. vagus*	*An. varuna*	*An. barbirostris*	*An. jamesii*	*An. nigerrimus*	*An. barbumbrosus*	*An. pseudojamesi*
Tank margin	+	+						+	+	
Waste water collection	+		+			+		+		
Water storage tank	+									
Field canal	+	+						+		
Main canal	+									
Paddy field	+	+				+		+	+	
Pond	+	+				+		+		
Built well (domestic)	+	+	+	+	+	+	+	+		+
Cemented tank	+									
Lagoon water collection	+									
Burrow pit	+		+	+	+	+	+	+		+
Rain water pool	+							+		

**Table 4:** Density and distribution of anopheline mosquito species encountered.

Mosquito species	Density (%)	Distribution (%)
*An. subpictus*	96.2	100.0
*An. varuna*	0.19	16.7
*An. nigerrimus*	0.75	66.7
*An. vagus*	0.03	16.7
*An. pallidus*	0.01	25.0
*An. peditaeniatus*	1.47	41.7
*An. jamesii*	0.005	16.7
*An. pseudojamesi*	0.005	16.7
*An. barbirostris*	1.23	41.7
*An. barbumbrosus*	0.1	16.7

**Table 5:** Correlation between anopheline densities climate variables.

Lag period (Months)	*Climatic variable *					
	*Rainfall*		*Temperature*		*Humidity*	
	All Anopheline	*An. subpictus*	All Anopheline	*An. subpictus*	All Anopheline	*An. subpictus*
*Hand collection*						
0	-0.178 (0.392)	-0.178 (0.392)	0.256 (0.215)	0.253 (0.221)	-0.265 (0.199)	-0.261 (0.206)
1	-0.167 (0.434)	-0.158 (0.459)	0.128 (0.549)	0.120 (0.575)	-0.071 (0.740)	-0.063 (0.767)
2	-0.105 (0.632)	-0.094 (0.667)	-0.067 (0.758)	-0.079 (0.719)	0.134 (0.540)	0.146 (0.506)

*Window trap collection*						
0	0.018 (0.932)	-0.111 (0.598)	-0.167 (0.424)	-0.167 (0.426)	0.167 (0.424)	0.096 (0.647)
1	0.156 (0.468)	0.215 (0.313)	-0.249 (0.240)	-0.302 (0.151)	0.207 (0.333)	0.254 (0.230)
2	0.104 (0.637)	0.209 (0.337)	-0.182 (0.405)	-0.267 (0.218)	0.192 (0.379)	0.337 (0.116)

*Cattle-baited trap collection*						
0	0.310 (0.132)	0.117 (0.576)	-0.278 (0.178)	-0.257 (0.215)	0.220 (0.290)	0.212 (0.309)
1	-0.034 (0.874)	-0.087 (0.686)	-0.292 (0.166)	-0.375 (0.071)	0.231 (0.277)	0.271 (0.199)
2	0.097 (0.659)	0.224 (0.303)	-0.246 (0.259)	-0.437^*∗*^ (0.037)	0.211 (0.334)	0.368 (0.084)

*Cattle-baited net collection*						
0	0.087 (0.680)	-0.034 (0.871)	-0.259 (0.211)	-0.272 (0.188)	0.227 (0.274)	0.164 (0.433)
1	0.222 (0.297)	0.285 (0.177)	-0.234 (0.271)	-0.314 (0.135)	0.298 (0.158)	0.339 (0.106)
2	0.147 (0.504)	0.241 (0.269)	-0.091 (0.679)	-0.196 (0.370)	0.181 (0.408)	0.325 (0.130)

*Larval survey*						
0	0.416^*∗*^ (0.039)	0.411^*∗*^ (0.041)	-0.368 (0.071)	-0.395 (0.051)	0.442^*∗*^ (0.027)	0.465^*∗*^ (0.019)
1	0.28 (0.177)	0.347 (0.097)	-0.283 (0.181)	-0.344 (0.100)	0.457^*∗*^ (0.025)	0.514^*∗*^ (0.010)
2	-0.033 (0.881)	0.059 (0.788)	-0.104 (0.636)	-0.176 (0.423)	0.171 (0.434)	0.238 (0.275)

P values in the parentheses.

*∗* Significant relationship.

**Table 6:** Physicochemical characteristics (mean ± SE, range) of breeding habitats in the district of Mannar.

Breeding places	Temperature (°C)	DO (mg/l)	pH (25°C)	Conductivity (*μ*s/cm)	Salinity (mg/l)	TDS (mg/l)	Turbidity (NTU)
Tank margin	31.71 ± 0.07a,d	5.84 ± 0.06a	7.94 ± 0.04a,c	1070.3 ± 26.7b,c	527.2 ± 15.4b	715 ± 18.6b	30.53 ± 1.82b
	(29.8 - 32.8)	(3.8 - 7.1)	(7.09 - 8.74)	(539 – 1763)	(244 – 764)	(162 – 986)	(3.72 - 76.8)
Waste water collection	31.70 ± 0.10a	3.45 ± 0.15a,c	7.87 ± 0.05a,c	3204 ± 294b	1347 ± 103b	1795 ± 136c,d	36.32 ± 5.85b
	(29.6 - 32.7)	(1.85 - 5.88)	(7.05 - 8.86)	(1068 – 7843)	(559 – 2746)	(722 – 3283)	(1.38 – 232)
Water storage tank	31.38 ± 0.16a,b	5.16 ± 0.15a,b,c	7.81 ± 0.11a,c,d	1403 ± 199b	629 ± 40.5b	744.8 ± 26.9b,c	0.74 ± 0.14b
	(30.8 - 31.7)	(4.81 - 5.71)	(7.48 - 8.12)	(1047 – 2175)	(538 – 729)	(673 – 816)	(0.42 - 1.21)
Field canal	31.43 ± 0.08a,d	5.54 ± 0.04a	7.62 ± 0.04a,d	1465 ± 115b,c	759.1 ± 66.2b	1058.4 ± 81.9b,e	43.35 ± 5.91b,c
	(30.2 - 31.9)	(5.06 - 5.98)	(7.28 - 7.96)	(933 – 2387)	(329 – 1349)	(600 -1864	(12.38 - 109
Main canal	31.4 ± 0.09a	5.0 ± 0.09a	7.95 ± 0.06a,c	10481 ± 1795a	5656 ± 1106a	6613 ± 1248a	15.31 ± 1.33a
	(30.1 - 32.1)	(4.02 - 5.9)	(7.25 - 8.64)	(1836 – 25100)	1033 - 14630	1323 - 16930	(4.83 - 28.8)
Paddy field	31.90 ± 0.08d,e	5.27 ± 0.08c	7.58 ± 0.05d	1656 ± 84.7b,c	905.7 ± 63.5b	1172.7 ± 72.1b,e	633.1 ± 83.8b,c
	(30.5 - 32.8)	(4.29 - 5.95)	(6.55 - 7.96)	(1003 – 2847)	(522 – 1575)	(735 – 1974)	(23.5 – 1928)
Pond	31.31 ± 0.07a	5.60 ± 0.18a	8.21 ± 0.095a	4128 ± 683c	2253 ± 400b	2725 ± 447d,e	25.96 ± 2.54c
	(30.0 - 31.9)	(4.21 - 11.6)	(7.21 - 9.89)	(852 – 26739)	(426 -15622)	(605 – 17623)	(4.02 - 82.1)
Built well	30.64 ± 0.09b	4.61 ± 0.07b	7.73 ± 0.0413c	1830 ± 179b,d	887.8 ± 87.7b,d	1175 ± 122b	4.52 ± 0.69 b
	(29.0 - 32.3)	(2.97 - 5.59)	(7.15 - 8.53)	(521 – 7243)	(219 – 3562)	(320 – 4537)	(0.26 - 24.9)
Cemented tank	30.60 ± 0.09b	5.03 ± 0.13b	7.93 ± 0.08a,c	2882 ± 233b,c	1175 ± 143b	1610 ± 178b,d	1.254 ± 0.113b,c
	(30.1 - 31.6)	(4.17 - 5.94)	(7.33 - 8.47)	(1172 – 3986)	(429 – 1983)	(712 – 2753)	(0.34 - 1.97)
Lagoon water collection	31.24 ± 0.19a,b	5.68 ± 0.53a	8.25 ± 0.16a	34734 ± 1974d	21105 ± 1344d	23156 ± 1462f	22.48 ± 2.35e
	(30.0 - 32.4)	(4.13 - 10.35)	(7.33 - 9.15)	(26000 – 45900)	(15600 – 28700)	(17060 – 30920)	(11.9 - 37.5)
Burrow pit	32.06 ± 0.15a,e	5.76 ± 0.09a	7.66 ± 0.07a,c	1458.4 ± 87.4b,c	589.2 ± 32.2b	786.8 ± 6.88b,e	56.85 ± 3.27b,c
	(31.2 - 32.7)	(5.33 - 6.13)	(7.29 - 7.89)	(1205 – 1865)	(453 – 785)	(776 – 848)	(34.14 - 75.3)
Rain water pool	31.59 ± 0.12a	4.28 ± 0.076a	8.65 ± 0.1b,d	7795 ±473a	3473 ± 282c	4917 ± 340a	29.77 ± 2.17a
	(30.2 - 32.4)	(3.28 - 4.93)	(8.03 - 9.89)	(4929 – 14720)	(2023 – 8283)	(2973 – 9744)	(17.73 - 59.22)

Note: values are given to the nearest significant decimal. Different superscript letters in a row show significant differences (*P* < 0.05) indicated by Tukey's multiple comparison after Kruskall-Wallis test.

## Data Availability

Results included in the manuscript were based on a work carried out for a postgraduate degree. Part of these results is included in the thesis submitted to the University of Kelaniya, Sri Lanka.
